# Ultrasound Shear Wave Simulation of Breast Tumor Using Nonlinear Tissue Elasticity

**DOI:** 10.1155/2016/2541325

**Published:** 2016-05-16

**Authors:** Dae Woo Park

**Affiliations:** ^1^Department of Biomedical Engineering, University of Michigan, Ann Arbor, MI 48105, USA; ^2^Department of Mechanical Engineering, University of Michigan, Ann Arbor, MI 48105, USA

## Abstract

Shear wave elasticity imaging (SWEI) can assess the elasticity of tissues, but the shear modulus estimated in SWEI is often less sensitive to a subtle change of the stiffness that produces only small mechanical contrast to the background tissues. Because most soft tissues exhibit mechanical nonlinearity that differs in tissue types, mechanical contrast can be enhanced if the tissues are compressed. In this study, a finite element- (FE-) based simulation was performed for a breast tissue model, which consists of a circular (*D*: 10 mm, hard) tumor and surrounding tissue (soft). The SWEI was performed with 0% to 30% compression of the breast tissue model. The shear modulus of the tumor exhibited noticeably high nonlinearity compared to soft background tissue above 10% overall applied compression. As a result, the elastic modulus contrast of the tumor to the surrounding tissue was increased from 0.46 at 0% compression to 1.45 at 30% compression.

## 1. Introduction

Pathological changes such as growth of malignant tumors in soft tissues result in increasing tissue stiffness and this produces elasticity contrast of tumors to the surrounding healthy tissues [1–3]. Sarvazyan et al. [4] introduced shear wave elasticity imaging (SWEI) for noninvasive diagnosis of changes in tissue mechanical properties. The propagation speed of the shear wave is directly related to the underlying tissue shear modulus. Shear waves in soft tissues can be generated by either direct mechanical vibration [5] or transient ultrasound (US) radiation force excitation [4, 6, 7]. The local tissue shear modulus can be reconstructed from the displacement field of shear waves using an inversion of the Helmholtz equation [6, 7] or time-of-flight (TOF) including random sample consensus [8] and the radon sum [9]. With the continued progress of technology and system development, SWEI has been investigated for diagnosing some diseases, mainly for detecting breast cancer [10] and liver cirrhosis [11, 12]. In some cases, pathologic characteristic changes of progressed malignant lesions have been successfully differentiated by SWEI from the normal surrounding soft tissues. However, SWEI may not be sensitive enough to detect small stiffness changes, especially in early stages of pathological changes [13].

It is known that most soft tissues exhibit significant strain hardening and elastic modulus of tissue can no longer be considered constant at large deformation [14]. In addition, the strain hardening varies for different tissue types since each tissue type has specific nonlinear elastic parameters [15]. The nonlinear characteristics of breast fibrosis and prostate cancer tissues have been demonstrated in their* ex vivo* mechanical measurements [3, 16] and these lesions could be differentiated better from surrounding normal tissues when being compressed. The tissue nonlinearity has been applied to compressional US elasticity imaging and an inclusion was detected with improved contrast and contrast-to-noise ratio* in vitro* and* in vivo* animal study [17]. The SWEI combined with an external compression was proposed and nonlinear shear modulus parameters have been investigated using* in vitro* phantom and* ex vivo* liver samples [18]. This nonlinear SWEI approach has been applied to* ex vivo* canine livers measurements and the increase of shear wave speed was presented with an increase of hepatic pressure [19]. In our previous study [20], the nonlinear characterization of tissue stiffness changes was demonstrated through* in vitro* phantom measurements using SWEI in combination with externally applied force. We found that the elastic modulus contrast of the inclusion to the surrounding phantom block was increased with an increase of the applied force.

In this study, the feasibility of SWEI combined with external compression for breast tumor detection was investigated using* in silico* finite element (FE) simulation. A FE hyperelastic breast tissue with a tumor model was designed. The shear modulus of the model was estimated at each compression level from 0% to 30% using TOF-based algorithms [9]. The elastic modulus contrast of the target tumor compared to surrounding tissue was calculated and analyzed over the compression levels.

## 2. Materials and Methods

### 2.1. Finite Element Modeling

**Figure 1:**
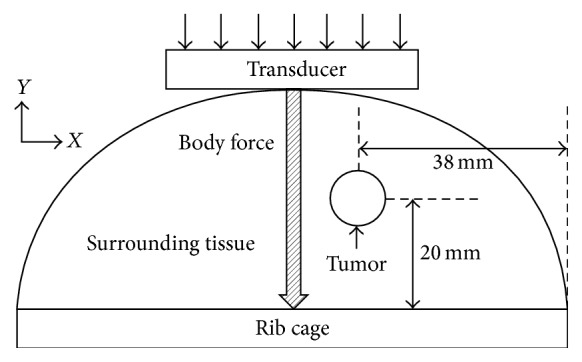
Schematic diagram of the breast tissue model with dimensions and boundary conditions for finite element simulations.

A 2D FE breast tissue model was developed using a commercially available software package (ABAQUS V6.13 Simulia, Dassault Sytèmes, RI, USA.).  Figure 1 shows the schematic diagram of the breast tissue model with dimensions and boundary conditions for FE simulations. A semiellipse shape of the breast model, which was 100 mm in diameter and 40 mm in height, was designed. The shape of the tumor field was assumed to be a circle with 10 mm diameter. The tumor was located 20 mm from the bottom surface and 38 mm from the right side of the model, as shown in Figure 1. The breast model was meshed using 2D triangular plane strain elements. The bottom boundaries of the breast tissue were fixed in all directions by a sternum (rib cage) to prevent any bulk motion from the local body force excitation for shear wave generation. A displacement boundary condition was applied on the top surface of the breast tissue by a rigid transducer of 45 × 14 mm. The contact between the surface of the transducer and top surface of the breast tissue was modeled as frictionless based on an assumption that ultrasound gel exerts no friction. To simulate the acoustic radiation force excitation, body forces were applied downward over an area of 3 mm width through entire depth of the FE model for 180 *μ*s to create planar shear waves at each compression level from 0% to 30%, which was calculated in the middle of the breast model. No shear wave attenuation was considered within the scope of the study. The *Y* displacements of shear waves over time were extracted over the entire *X* (across the body force direction) extent of the mesh. The temporal and spatial resolution of FE simulation was 125 *μ*sec and 0.2 × 0.2 mm, respectively.

### 2.2. Nonlinear Material Parameters

The hyperelastic material model (polynomial strain-energy function) was employed for the tumor and surrounding tissue. The polynomial strain-energy function [21], which is widely used in modeling soft tissues such as breast, is defined as

(1) $$\begin{array}{c} U=\overset{N}{\underset{i+j=1}{\sum }}C_{ij}{\left(\;I_{1}-3\;\right)}^{i}{\left(\;I_{2}-3\;\right)}^{j}+\overset{N}{\underset{i=1}{\sum }}\frac{1}{D_{i}}{\left(\;J_{\text{el}}-1\;\right)}^{2i}, \end{array}$$

where *U* is a selected strain energy function and *I*_1_ and *I*_2_ are the first and second strain invariants, respectively. *J*_el_ is the elastic volume strain, and *D*_*i*_ is a compressibility coefficient. *N* refers to the order of the model and *N* = 2 in this study; *C*_*ij*_ represents the hyperelastic material parameters, which determine the intrinsic nonlinear elastic properties of the tissues. The hyperelastic material parameters, *C*_*ij*_, were chosen from previous* ex vivo* breast tissue measurements [22]. Poisson's ratio was selected as 0.495 based on incompressible tissue assumptions.  Table 1 presents the dimensionless nonlinear parameters of the normal breast tissue and the malignant tumor.

### 2.3. SWEI-Modulus Reconstruction

To remove wave reflections near the tumor area, a 1D directional filter [23], which identifies the backward-moving shear waves in *X* direction and eliminates the waves in the frequency domain, was applied to the *Y* displacements versus time data. TOF methods were used to determine the shear wave speed from *Y* displacements field by estimating shear wave arrival time at each *X* position and calculating the slope of the position versus time data [9]. The slope was calculated using data from the positions within a 3 mm kernel in *X* direction that was stepped across the *X* range. Shear wave speeds were reconstructed using a 3 mm kernel in *Y* direction and then smoothed using a 2 mm × 2 mm median filter.

The shear modulus was determined from the estimated shear wave speeds as

(2) $$\begin{array}{c} G=\rho c^{2}, \end{array}$$

where *ρ* is density of medium and *c* is speed of the shear wave. Each shear modulus for the tumor and the surrounding tissue was spatially averaged within the area of the tumor size. The average shear moduli in the tumor and surrounding tissue were compared at each compression level up to 30%.

## 3. Results

**Figure 2:**
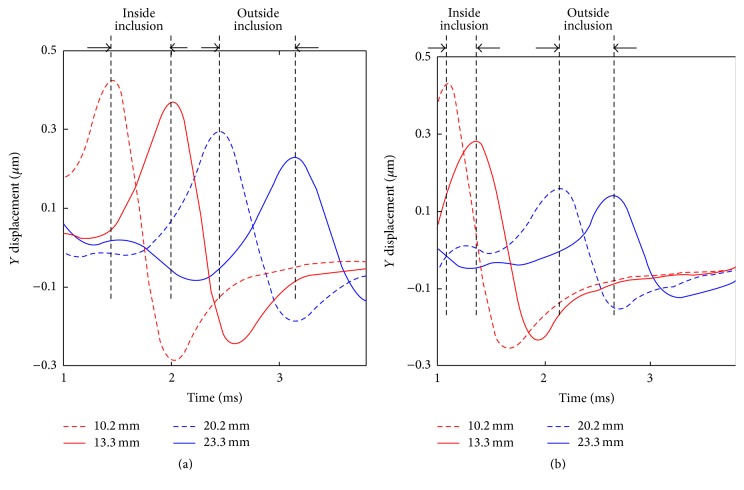
*Y* displacements versus time profiles of a finite element tissue model at (a) 0% and (b) 30% compression at four *X* positions.

Figure 2 shows *Y* displacements versus time profiles of a FE tissue model at (a) 0% and (b) 30% compression at four *X* positions. Two *X* positions (10.2 mm and 13.3 mm from the middle of breast), marked with red dashed line and red solid line, were selected from inside of the tumor. The other two *X* positions (20.2 mm and 23.3 mm from the middle of breast), marked with blue dashed line and blue solid line, were chosen from outside of the tumor. For both inside and outside of the tumor, distance between two *X* positions was kept the same for 3.1 mm. Higher shear wave speed was estimated inside the tumor at 0% compression. The rate of shear wave speed increase was distinctively higher by factor of 1.3 inside the inclusion from 0% to 30% compression.

**Figure 3:**
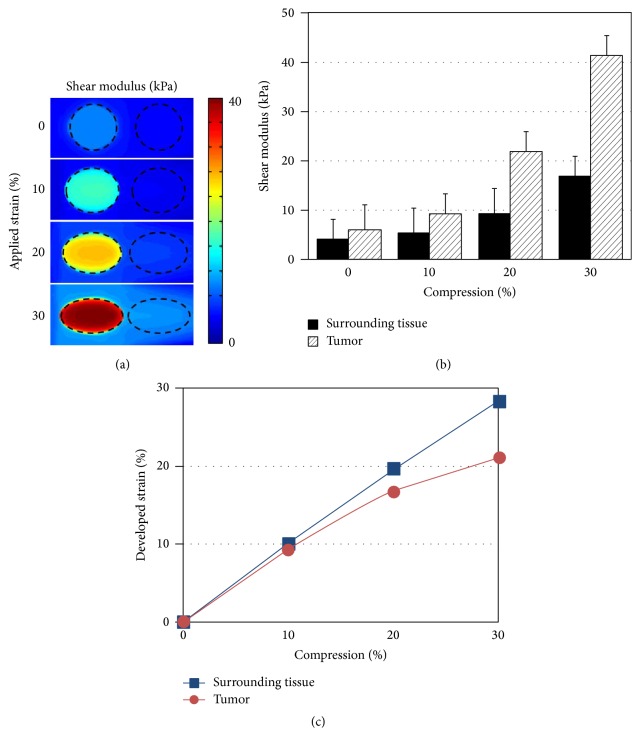
Shear wave elasticity imaging on a finite element breast tissue model. (a) Reconstructed shear modulus map near a tumor region with 0%, 10%, 20%, and 30% compression (applied strains). Black dashed circle represents the boundary of a tumor and surrounding tissue. (b) Average shear modulus versus compression plot for the tumor and surrounding tissue. (c) Average developed strain versus compression plot for the tumor and surrounding tissue.

Figure 3(a) presents reconstructed shear modulus maps near a tumor region in a FE breast tissue model with 0%, 10%, 20%, and 30% compression. The black dashed circle represents the boundary of a tumor and surrounding tissue. The increase of shear modulus for both tumor and surrounding tissue was observed with increase of compression level from 0% to 30%. The shear modulus calculated by TOF was spatially averaged in the tumor and surrounding tissue depicted in Figure 3(a). In Figures 3(b) and 3(c), the average shear moduli and developed strains for the tumor (diagonal stripe pattern bar) and surrounding tissue (solid bar) versus compression levels up to 30% are compared. The error bar represents the standard deviation of spatially averaged shear modulus for the tumor and surrounding tissue. At 0% compression, the average shear modulus of the tumor was 6 kPa and it sharply increased up to 41 kPa at 30% compression. The average strain of the tumor was 9% at 10% compression and it increased up to 21% at 30% compression. On the other hand, the average shear modulus of surrounding tissue was 4 kPa at 0% compression and it almost linearly increased up to 17 kPa at 30% compression. The average strain of the surrounding tissue was 10% at 10% compression and it increased up to 28% at 30% compression.

Shear modulus contrast *C* was calculated for different compression levels of 0%, 10%, 20%, and 30%, which is defined as

(3) $$\begin{array}{c} C=\left|\;\frac{G_{\text{tumor}}-G_{\text{surr}}}{G_{\text{surr}}}\;\right|, \end{array}$$

where *G*_tumor_ and *G*_surr_ are the corresponding average shear modulus in the tumor and surrounding tissue, respectively. The shear modulus contrasts were 0.46, 0.71, 1.33, and 1.45 at 0%, 10%, 20%, and 30% compressions, respectively. Thus, the elastic modulus contrast of the tumor to the surrounding tissue was enhanced by the compression, and this allows much better identification of the tumor.

## 4. Discussion

The enhancement of the elastic modulus contrast for SWEI was demonstrated through a FE simulation using a new approach of nonlinear SWEI in combination with applying external compression. The shear modulus of a breast tumor exhibited noticeably high nonlinearity compared to soft background tissue above 10% overall applied compression. As a result, the tumor became more visible with increased contrast as shown in the reconstructed shear modulus map in Figure 3(a).

The rate of shear modulus increase was small, similar between the tumor and surrounding tissue for the applied compression level below 10% as shown in Figure 3(b). The surrounding tissue plays a significant role in balancing the force under relatively small applied compression. A large portion of the applied force must have been absorbed by the large, soft surrounding tissue, relieving exerting stress on the small tumor. The rate of shear modulus increase in the tumor became higher than the surrounding tissue as the overall applied compression increased from 10% to 30%. This is because the stress inside the target tumor increased more once the surrounding background tissue became stiff over the 10% compression.

There are two reasons we limited the compression level to the maximum of 30% in this study. Firstly, the developed strains inside the tumor and surrounding tissue that were calculated from *Y* displacements were 21% and 28%, respectively, with 30% overall applied compression as shown in Figure 3(c). Such levels of developed strains were high enough for breast tissues to exhibit strong nonlinearity. Secondly, the 30% applied compression remains within the practical range for the clinical application since most soft tissues start exhibiting nonlinearity over 5–10% strain [14] and the elastic modulus contrast enhancement can be achieved without causing any pain by applying excessive deformation on the skin.

In this study, the acoustic radiation force excitation was simulated using body forces applied through entire depth of the FE model. In actual SWEI, two quasi-planar shear waves, which are not parallel to each other, are normally generated using successive focusing ultrasonic beam at different depths [7]. To compute the elastic modulus contrast of the tumor to surrounding tissues under idealistic conditions, parallel planar shear waves were generated using the body forces in the FE simulation. The shear modulus computation depends on the shape of shear wave front [7] and the elastic modulus contrast of tissues may change if it is computed using the quasi-planar shear waves. Thus, the effect of quasi-planar shear waves on the elastic modulus contrast needs to be further investigated by the FE model using the actual acoustic radiation force excitation.

The practical application of the nonlinear SWEI approach would be identifying lesions by using different nonlinearity characteristics for different tissue types. In breast, fatty tissues exhibit almost linear stress-strain relationship while disease tissues such as fibrous, ductal, and intraductal tumors have their specific tissue nonlinearity characteristics [16]. The prostate tumor also has its specific tissue nonlinearity that is differentiated from surrounding normal tissues [3]. The shear modulus contrast of lesions would vary due to different nonlinearity characteristics for different tissue types. Investigating relations between the shear modulus contrast and tissue nonlinear parameters in different tissues would help for nonlinear SWEI application to potentially classify disease tissues. The nonlinear SWEI approach may be able to differentiate lesions by applying enough compression that can create a large shear modulus contrast between different disease tissue types and this can be applied to breast mammography to improve accuracy of diagnosis [24].

There are several limitations of our FE simulation approach. Firstly, the elastic modulus contrast to surrounding tissue computed from the 2D FE model may not represent the elastic modulus contrast of the 3D tissue. The tissue elasticity usually varies in the out-of-plane direction as well as in a 2D plane. The elastic modulus contrast may also change according to locations of compression in out of plane direction. Secondly, the shear wave attenuation commonly occurs in human tissues due to viscosity [25], and this was not considered. The amplitude of the shear wave decreases with the propagation due to shear wave attenuation [25]. Thus, the signal-to-noise ratio also decreases with respect to the lateral distance from the location of acoustic force excitation [25]. This may affect the shear modulus computation and the elastic modulus contrast of the tumor to surrounding tissues. Thirdly, the shear modulus was computed from the displacement fields of shear waves without including ultrasonic noise such as jitter. The jitter commonly exists in ultrasonically tracked displacement data, and substantial filtering is necessary before processing the data [26]. The filtering may affect shear modulus reconstruction especially at the boundary of a tumor in the tissue model. Therefore, these limitations should be considered in a future version of the FE model for nonlinear SWEI simulation.

The nonlinear SWEI approach has several limitations to be considered for practical use of this technology. Firstly, mechanical compression of tissue is necessary for the nonlinear SWEI and this limits its applications to areas where clear physical access can be achieved [27]. Secondly, consistent forces are necessary to maintain the mechanical compression of tissue. A target inclusion can be out of scanning plane with excessive compressions, and this may change the mechanical contrast to background tissues. Thirdly, the strain generation inside the target inclusion is related to the stress distribution that depends on the applied force and surrounding anatomy [27]. Thus, the internal strains in the inclusion may not develop high enough to exhibit nonlinearity if the target inclusion is located too deep from the skin. Due to these limitations, the application of nonlinear SWEI approach would be restricted to skin or organs close to skin surface.

In future studies, we will perform the nonlinear FE simulation by changing the boundary conditions of tissue model such as tumor size and tumor location. The elastic modulus contrast to surrounding tissue would vary according to the change of stress and strain distributions in tissues. The stress and strain distributions in tissues during compression are closely related to the boundary conditions. In this study, the average stresses of tumor and surrounding tissues were 26 kPa and 14 kPa at 30% compression, respectively. The ideal shear modulus contrast computed using the stresses and strains of the tumor and surrounding tissues was 1.45 at 30% compression. Therefore, conducting the FE simulation by varying the boundary conditions would be necessary to establish the nonlinear SWEI application for various tumor types.

## 5. Conclusion

The* in silico* FE simulation of nonlinear SWEI combined with external deformation demonstrated remarkable enhancement of the elastic modulus contrast of breast tumor to surrounding tissue. The encouraging results from* in silico* FE simulation warrant further investigation of this technique using disease relevant tissues* ex vivo* and eventually* in vivo*. With further development and evaluation, the nonlinear SWEI may allow noninvasively assessing and monitoring subtle stiffness changes in breast tissues due to the growth of malignant tumors at early disease stage.

## Figures and Tables

**Table 1:** Hyperelastic parameters of malignant tumor and benign breast tissue.

Variable	Malignant tumor	Benign breast tissue
*C*_10_	1.41 × 10^−3^	0.375 × 10^−3^
*C*_01_	1.41 × 10^−3^	0.375 × 10^−3^
*C*_11_	17.1 × 10^−2^	0.0256 × 10^−1^
*C*_20_	1.66 × 10^−2^	0.0283 × 10^−2^
*C*_02_	1.66 × 10^−2^	0.0283 × 10^−2^
